# Health: Its Friends and Its Foes

**Published:** 1862-08

**Authors:** 


HEALTH: ITS FRIENDS AND ITS FOES. By R. D. MUSSEY, M.D., LL.D., late Professor of Anatomy and Surgery at Dartmouth College, N.H., and of Surgery in the Medical College of Ohio; Fellow of the American Academy of Arts and Sciences, etc., etc. Boston: GOULD & LINCOLN. 1862.

This is a neatly printed volume of 368 pages, embracing the consideration of a variety of interesting and important topics.

The author is one of the oldest and most eminent of the living members of our profession; and, though he has written more directly for the benefit of the public at large, yet every physician will find himself amply remunerated for the time spent in a perusal of the work.

It is, in fact, a very valuable work on personal hygiene; embodying the personal experience and extended observations of one of the most erudite men of our time, on the following subjects:—The Corset—Clothing—Boots and Shoes.

Ventilation—Light—Sleep—Exercise—Bathing.

Alcohol—Its Effects in Health and Disease.

Tobacco—Its Influence upon Life and Health.

Tea and Coffee.

Casper Hauser.

Organic Sympathies.

Man by Nature a Vegetable Eater.

Diseases of the Teeth, and of Wild Animals.

Man Omnivorous by Practice—Gluttony, etc.

Quantity of Food—Economy of Vegetable Diet, etc.

Vegetable Food Sufficient for Man—Moral and Intellectual Effects of Vegetable Diet, etc.

Objections to Vegetarianism—Cherokees, etc.

Vegetable Diet—Illustrative Cases.

Injudicious Diet and Disease—Cases.

Vegetable Diet as a Remedy—Cases.

Ophthalmia—Deaths from Eating—Distillery-fed Pork—Intemperance in Eating and Drinking.

Apoplexy—Palsy—Epilepsy.

Epilepsy—Dyspepsia.

Constipation—Colds.

Blood Poisoning—Parasites.

My Own Experience.

Milk and Vegetable Feeding for Surgical Operations.

Length of Life.

All these subjects are discussed in a plain, but chaste and pleasing style.

We cordially commend the work to the public and the profession.

